# Dietary lysophospholipids supplementation inhibited the activity of lipolytic bacteria in forage with high oil diet: an *in vitro* study

**DOI:** 10.5713/ajas.19.0850

**Published:** 2020-02-25

**Authors:** Hanbeen Kim, Byeongwoo Kim, Seongkeun Cho, Inhyuk Kwon, Jakyeom Seo

**Affiliations:** 1 Department of Animal Science, Life and Industry Convergence Research Institute, Pusan National University, Miryang 50463, Korea; 2 EASY BIO, Inc., Seoul 06253, Korea

**Keywords:** Lysophospholipids, Emulsifier, Feed Additive, Rumen Fermentation, Microbial Diversity

## Abstract

**Objective:**

The objective of this study was to evaluate the effects of lysophospholipids (LPL) supplementation on rumen fermentation, degradability, and microbial diversity in forage with high oil diet in an *in vitro* system.

**Methods:**

Four experimental treatments were used: i) annual ryegrass (CON), ii) 93% annual ryegrass +7% corn oil on a dry matter (DM) basis (OiL), iii) OiL with a low level (0.08% of dietary DM) of LPL (LLPL), and iv) OiL with a high level (0.16% of dietary DM) of LPL (HLPL). An *in vitro* fermentation experiment was performed using strained rumen fluid for 48 h incubations. *In vitro* DM degradability (IVDMD), *in vitro* neutral detergent fiber degradability, pH, ammonia nitrogen (NH_3_-N), volatile fatty acid (VFA), and microbial diversity were estimated.

**Results:**

There was no significant change in IVDMD, pH, NH_3_-N, and total VFA production among treatments. The LPL supplementation significantly increased the proportion of butyrate and valerate (Linear effect [Lin], p = 0.004 and <0.001, respectively). The LPL supplementation tended to increase the total bacteria in a linear manner (p = 0.089). There were significant decreases in the relative proportions of cellulolytic (*Fibrobacter succinogenes* and *Ruminococcus albus*) and lipolytic (*Anaerovibrio lipolytica* and *Butyrivibrio proteoclasticus*) bacteria with increasing levels of LPL supplementation (Lin, p = 0.028, 0.006, 0.003, and 0.003, respectively).

**Conclusion:**

The LPL supplementation had antimicrobial effects on several cellulolytic and lipolytic bacteria, with no significant difference in nutrient degradability (DM and neutral detergent fiber) and general bacterial counts, suggesting that LPL supplementation might increase the enzymatic activity of rumen bacteria. Therefore, LPL supplementation may be more effective as an antimicrobial agent rather than as an emulsifier in the rumen.

## INTRODUCTION

The addition of a lipid source has been used to increase energy density in cattle diets because of the higher energy content of lipids compared with carbohydrate and protein on a weight basis. Conversely, the use of excessive lipids (6% to 7% of the dietary dry matter [DM]) in the ruminant diet might decrease DM intake, which could negate the increased energy density [1].

Lysophospholipids (LPL) are monoacyl-derivatives of phospholipids resulting from the action of phospholipase A1 or A2 [2] and can improve the digestion and absorption of lipids. The LPL has been used as a feed additives to improve the production performance of nonruminant animals and has consistently shown an increase in feed efficiency and nutrient utilization [3–6].

In ruminant nutrition, to our knowledge, few studies have investigated the effects of LPL supplementation on ruminants [7–9]. Jenkins et al [7] reported that the addition of phospholipids had different effects on ruminal fermentation characteristics depending upon the source of the LPL with no positive effect on fiber digestion in an *in vitro* system. However, Sontakke et al [8] observed that supplementation of LPL obtained from rice bran could increase *in vitro* organic matter (OM) digestibility when 6% of LPL was supplemented in the diet. Lee et al [9] reported an increase in milk yield and milk components (amounts of protein and lactose in milk) with increasing levels of LPL supplementation (0.05% and 0.075% of dietary DM). Furthermore, emulsifying agents (e.g., non-ionic surfactants) have been reported to improve the enzymatic activities of protease and cellulase in the rumen, even though they are not components of LPL [10].

To our knowledge, no study has investigated the effects of LPL on rumen fermentation in fiber containing a high lipid source, and we questioned whether LPL supplementation could prevent the negative effects resulting from high oil content, such as decreased fiber digestion and volatile fatty acid (VFA) production. This study was conducted to evaluate the effects of LPL supplementation on rumen fermentation characteristics, ruminal degradability, and microbial diversity in a forage-based diet with high oil content in an *in vitro* system.

## MATERIALS AND METHODS

Animal use and experimental protocols were reviewed and approved by the Animal Research Ethics Committee of Pusan National University (PNU-2019-2239).

### Preparation of experimental diets and chemical analysis

An experimental total mixed ration was prepared using annual ryegrass, corn oil (C8267, Sigma-Aldrich Co., St. Louis, MO, USA), and LPL, and the chemical compositions of the experimental diets are shown in Table 1. Before chemical analysis was conducted, feed ingredients and experimental diets were dried at 60°C for 96 h and ground through a cyclone mill (Foss Tecator Cyclotec 1093, Foss, Hillerød, Denmark) fitted with a 1 mm screen. The DM (#934.01), crude protein (CP, #976.05), ether extract (EE, #920.39), acid detergent fiber (#973.18), and ash (#942.05) were analyzed by AOAC international methods [11]. The CP was calculated by multiplying the nitrogen content by 6.25, and total nitrogen was measured using the Kjeldahl method with a nitrogen combustion analyzer (Leco FP-528 Leco, St. Joseph, MI, USA). Neutral detergent fiber (aNDF) and lignin were analyzed [12] to determine the fiber content. Heat-stable amylase (α-amylase) was used to estimate aNDF and was expressed inclusive of residual ash. Non-fibrous carbohydrate (NFC) of the experimental diets was estimated as follows:

NFC=[100-ash-EE-CP-aNDF].

### Experimental treatments

A commercial LPL (Lipidol Ultra, Easy Bio Inc., Seoul, Korea) was used in this study as an emulsifier. According to the manufacturer, the LPL is hydrolyzed soy lecithin with the inclusion of phospholipids and free fatty acids. A complete randomized block design was used for the experiment, with treatment as the main effect. Four experimental treatments were used as follows: i) annual ryegrass (CON), ii) 93% annual ryegrass + 7% corn oil on a DM basis (OiL), iii) OiL diet supplemented with a low level (0.08% of dietary DM) of LPL (LLPL), and iv) OiL diet supplemented with a high level (0.16% of dietary DM) of LPL (HLPL).

### *In vitro* fermentation

*In vitro* fermentation was carried out using the rumen fluid collected from two cannulated Holsteins (body weight [BW] 450±30 kg) before the morning feed at the Center for Agriculture Research, Pusan National University, Korea. Animals were fed a diet consisting of 600 g/kg Timothy hay and 400 g/kg of a commercial concentrate mix. The rumen fluid was collected before the morning feeding time, mixed, transferred into a thermos bottle, and immediately transported to the laboratory. Rumen content was filtered through four layers of cheesecloth and mixed with 3× volume of *in vitro* rumen buffer solution [13] under strictly anaerobic conditions. Approximately 0.5 g of the ground experimental substrates were placed into pre-weighed nylon bags (R510, Ankom Technology, Fairport, NY, USA). All the bags were heat-sealed and transferred into empty 125 mL serum bottles. Three bottles were used per dietary treatment, and each bottle contained two bags. Then, 70 mL of rumen fluid and buffer mixture was transferred, accompanied by continuously flushing with O_2_-free CO_2_ gas. The bottles were sealed with butyl rubber stoppers and aluminum caps and incubated on a rotary shaker (JSSI-300T, JS Research Inc., Gongju, Korea) at 20 rpm for 48 h at 39°C. After incubation for 48 h, gas production, *in vitro* DM degradability (IVDMD), *in vitro* NDF degradability (IVNDFD), pH, ammonia nitrogen (NH_3_-N), and VFA concentrations were measured. Gas production was measured at 3, 6, 12, 24, and 48 h by using a pressure transducer (Sun Bee Instrument Inc., Seoul, Korea) as described by Theodorou et al [14]. Gas production profiles obtained during incubation were fitted to a simple exponential model [15], and the equation is as follows:

$$\begin{array}{ll} V_{T}=0 & (0\leq \text{T}\leq \text{L}),\\ V_{T}=V_{\text{max}}\times (1-{\text{e}}^{\left[-Kg\times (T-L)\right]}) & (\text{T}\geq \text{L}), \end{array}$$

where T is time (h), L is lag time (h), e is the exponential function, K_g_ is the fractional rate of gas production (h^−1^), V_max_ is the theoretical maximum gas production (mL) after the asymptote is reached, and V_T_ is gas produced at time T (mL). After incubation, the bottle caps were removed, and the bottles were fixed immediately on ice to stop the fermentation. The nylon bags were then removed from the bottles and rinsed under flowing water until the water ran clear. The washed bags were then dried at 60°C for 72 h and weighed to measure IVDMD. The NDF content of the weighed bags was assessed using a modified version of the micro-NDF method proposed by Pell and Schofield [16] to evaluate the IVNDFD. The pH of the culture fluid was measured using a pH meter (FP20, Mettler Toledo, Columbus, OH, USA). Sample fluid (5 mL) was centrifuged at 20,000×*g* for 20 min at 4°C, the supernatant was discarded, and the pellet was stored at −80°C until rumen microbial population analysis. The remaining culture fluid was then centrifuged at 15,000 ×*g* for 10 min at 4°C and stored at −20°C, until VFA and NH_3_-N analysis.

For the VFA analysis, 200 μL of the supernatant was diluted with 800 μL of ethyl alcohol anhydrous (4023-2304, Daejung Chemicals, Siheung, Korea) after 15 min of centrifugation at 20,000×*g*. VFA was measured via gas chromatography (Agilent 7890A, Agilent Technology, Santa Clara, CA, USA) equipped with a flame ionization detector and capillary column (Nukol Fused silica capillary column, 30 m×250 μm ×0.25 μm, Supelco Inc., Bellefonte, PA, USA). The temperature of the oven, injector, and detector was set at 90°C, 90°C to 200°C, and 230°C, respectively. Nitrogen was used as the carrier gas at a flow rate of 30 mL/min. The NH_3_-N concentration was analyzed with several modifications [17]. After 2 μL of the supernatant was mixed with 100 μL of phenol color reagent (50 g of phenol, 0.25 g of sodium nitroferricyanide, and 1 L of distilled water) and alkali hypochlorite (25 g of sodium hydroxide, 16.8 mL of sodium hydroxide, and 1 L of distilled water), the mixture was incubated in a water bath at 37°C for 15 min. The ammonia concentration was determined by measuring the optical density at 630 nm using a microplate reader (iMARK, Bio-Rad, Hercules, CA, USA).

### Total DNA extraction and real-time polymerase chain reaction

Total DNA was extracted from the pellet stored at −80°C using the repeated bead beating plus column (RBB+C) method [18]. Genomic DNA was treated with RNase A and proteinase K and purified using columns from the DokDo-Prep Genomic DNA Kit (Elpis-Biotech, Daejeon, Korea). The concentration and purity of total DNA were measured using a NanoDrop (ND-1000, Thermo Fisher, Waltham, MA, USA).

Real-time polymerase chain reaction (PCR) assays were performed on a CFX 96 Touch system (Bio-Rad Laboratories, Inc., Hercules, CA, USA). Information on primer sequences for rumen microbes was collected from previous studies [19,20] and is presented in Table 2. Reactions were performed in triplicate, in reaction volumes of 20 μL, by using optical reaction plates sealed with optical adhesive film. Each reaction mixture contained 0.5 μL 10 mM dNTP Mix (BioFACT, Daejeon, Korea), 2 μL 10× buffer (BioFACT, Korea), 1 μL of genomic DNA diluted 10-fold, 1 μL forward primer (10 μM), 1 μL reverse primer (10 μM), 0.1 μL Taq polymerase (BioFACT, Korea), 1 μL Evagreen (SolGent Co., Ltd., Daejeon, Korea), and 13.4 μL PCR-grade water. Real-time PCR was carried out according to the manufacturer’s instructions, as follows: initiation for one cycle at 95°C for 10 min; 40 cycles each for denaturation at 95°C for 30 s, annealing at 60°C for 30 s, and elongation at 72°C for 30 s; and final elongation at 72°C for 5 min. Fluorescence was recorded at the end of each denaturation and extension step, and the specificity of the amplicon was confirmed via dissociation curve analysis of PCR end products by increasing the temperature at a rate of 1°C every 30 s, from 60°C to 95°C. For absolute quantification of each microbe, standard plasmids containing the respective target gene sequence were obtained by PCR cloning using each primer set described in Table 2. The copy number of each standard primer was calculated [21] and diluted in 10-fold serial dilutions. CFX manager software (Bio-Rad, USA) was used to compare microbe quantifications with the standard curve.

### Statistical analysis

Statistical analyses were performed using the PROC GLIMMIX procedure of SAS 9.3 (SAS Institute Inc., Cary, NC, USA). The fixed effect in this model was a treatment. Orthogonal contrast was used to analyze the difference between OiL and LPL (LLPL and HLPL) treatments, with a linear effect (Lin) when the level of LPL supplementation increased. Differences among treatments were compared using Tukey’s range test if a significant effect was observed. Statistical significance was declared at p<0.05, and a trend was speculated at 0.05 ≤ p<0.10.

## RESULTS

The effects of LPL on gas production and parameters are presented in Table 3. There was no difference in gas production among treatments until 6 h incubation. Gas production in CON was significantly higher at 12 h incubation and tended to be higher than that in other oil supplementation groups at 24 h (treatment effect [TRT], p = 0.004 and 0.052, respectively). Although final gas production in CON was significantly higher than that in OiL and LLPL, no difference was observed between CON and HLPL. No significant difference in V_max_ and K_g_ was detected among treatments (Table 3).

There was no significant change in IVDMD, pH, NH_3_-N, and VFA production among all treatments (Table 4), whereas corn oil supplementation had a negative effect on IVNDFD (TRT, p = 0.007). Regarding the proportion of individual VFAs, oil supplementation significantly decreased the proportion of acetate and the acetate to propionate (A:P) ratio (both, p<0.001) and increased the proportion of propionate, valerate, iso-valerate (TRT, p<0.0001, <0.0001, and = 0.032, respectively). Propionate proportion tended to decrease with increasing levels of LPL supplementation (Lin, p = 0.057), although there was no significance in the proportion of acetate and iso-butyrate. LPL supplementation significantly increased the proportion of butyrate (OiL vs LPL, p = 0.007; Lin, p = 0.004), valerate (OiL vs LPL, p<0.001; Lin, p<0.001), and iso-valerate (OiL vs LPL, p = 0.021; Lin, p = 0.037). The A:P ratio numerically increased with increasing levels of LPL supplementation (Lin, p = 0.105).

Regarding the microbial counts, although there was no significant change in the absolute value of total bacteria among treatments, LPL supplementation tended to increase total bacteria in a linear manner (Lin, p = 0.089) compared to OiL (Table 5). Absolute counts of ciliate protozoa and Fungi were not changed by oil supplementation among treatments, but Fungi was affected by LPL treatment (OiL-LPL, p = 0.048). In the relative proportion of each bacteria, fiber degrading bacteria (*Fibrobacter succinogenes* [*F. succinogenes*], *Ruminococcus albus* [*R. albus*], and *Ruminococcus flavefaciens* [*R. flavefaciens*]) was decreased by oil supplementation (TRT, *F. succinogenes*, p = 0.008; *R. albus*, p<0.001; and *R. flavefaciens*, p = 0.001). The relative proportion of *F. succinogenes* and *R. albus* was significantly decreased in a linear manner by LPL supplementation (Lin, *F. succinogenes*, p = 0.028; *R. albus*, p = 0.006). There was no significant change in *Butyrivibrio fibrisolvens*, whereas oil supplementation resulted in a significantly higher proportion of *Anaerovibrio lipolytica* (*A. lipolytica*) than that in the CON group (TRT, p<0.001). The highest proportion of *Butyrivibrio proteoclasticus* (*B. proteoclasticus*) was observed in the OiL group (TRT, p = 0.002), and LPL supplementation resulted in lower relative proportions of *B. proteoclasticus* and *A. lipolytica* than that in the OiL group (Lin, *B. proteoclasticus*, p = 0.003; *A. lipolytica*, p = 0.003).

## DISCUSSION

Oil supplementation can change rumen fermentation characteristics and decrease nutrient digestibility, which is mainly induced by a shift of microbial diversity [22,23]. Hence, the effect of fat supplementation on rumen fermentation is varied depending on the type and saturation of fat and the type of diets [23,24]. Matsuba et al [22] observed that supplementing 5% DM of soybean and coconut oils and 7.5% DM of palm oil had a negative influence on the proportion of *F. succinogenes* during *in vitro* rumen fermentation. Jenkins [25] suggested that the inclusion of fat in the rumen could have antimicrobial effects and also coat the feed particles, thereby inhibiting digestibility. The results from the present study showed that substituting 7% DM with corn oil in forage significantly decreased the proportion of fibrolytic bacteria (*F. succinogenes* and *R. albus*) and IVNDFD.

Previous studies have shown that the main positive effects of LPL supplementation on nonruminant animals were increase of apparent nutrient digestibility, thereby improving feed efficiency [4,6]. Conversely, Jenkins et al [7] reported that supplementation of lecithin (2% to 6% of dietary DM) decreased NDF and OM digestibility during *in vitro* rumen fermentation. Lee et al [9] also reported that LPL supplementation tended to decrease the apparent digestibility of DM and OM and induced a numerical decrease in the apparent digestibility of NDF, although they observed an increased milk yield resulting in increased feed efficiency. Considering the results of previous studies on ruminant, the effects of LPL as an emulsifier is unclear in the general ruminant diet. In addition, oil supplementation is one of the alternative strategies to increase energy density of diet when ruminant animal required high energy [22]. Therefore, in this study, we focused on the emulsifying ability of LPL on the rumen ecosystem when the ruminant diet was supplemented with a high lipid content. We hypothesized that LPL supplementation could prevent the negative effects of oil supplementation by emulsification of the supplemented oil. Contrary to our expectations, in the present study, there were no effects on total gas production and/or on the degradability of DM and NDF *in vitro* between OiL versus LPL groups; even the relative proportions of several cellulolytic bacteria (*F. succinogenes* and *R. albus*) significantly reduced with increasing levels of LPL supplementation. Lee et al [10] reported that Tween 80 (type of emulsifier) significantly increased the *in situ* DM disappearance in rice straw, although the emulsifier reduced the attachment of major rumen cellulolytic bacteria (*F. succinogenes*, *R. albus*, and *R. flavefaciens*). Similarly, Kamande et al [26] stated that the use of emulsifiers (Tween 60 and Tween 80) might enhance rumen microbial cellulase activities, inducing an increase in cellulose degradation rather than improving the attachment ability of fibrolytic bacteria. Considering previous results, it is possible that LPL supplementation could increase the enzymatic activity of cellulolytic bacteria similar to that by other emulsifiers (Tween 60 and Tween 80) in the rumen under high lipid conditions.

In the present study, oil supplementation decreased the proportion of acetate and A:P ratio and increased the proportion of propionate in total VFA; however, the total VFA concentration did not change. Getachew et al [27] reported an increase in propionate production and decrease in acetate production and A:P ratio with supplementation of corn oil in an *in vitro* system. Similarly, Pi et al [28] also observed that supplementation of 4% rubber seed oil and flaxseed oil significantly increased the proportion of propionate, whereas it decreased the proportion of acetate in dairy cattle. Generally, lipid sources entering the rumen are degraded into glycerol, sugars, and free fatty acids through hydrolysis of ester linkages by rumen lipolytic bacteria (e.g. *A. lipolytica*, *B. fibrisolvens*, and *B. proteoclasticus*) [29]. In the present study, oil supplementation significantly increased the relative proportion of *A. lipolytica*, one of the major rumen bacteria related to lipid hydrolysis, which might then increase glycerol and sugar that can be metabolized to VFA. It was previously reported that glycerol supplemented in the rumen could increase proportion of propionate at the expense of acetate proportion [30]. In addition, corn oil had high proportion of unsaturated fatty acids, and the unsaturated fatty acids could negatively affect ruminal fibrolytic bacteria, thereby decreasing A:P ratio [31]. Thus, we suggested that the changes between acetate and propionate proportion might be related to improved lipid hydrolysis.

In this study, LPL supplementation increased the proportion of butyrate, valerate, and iso-valerate but tended to decrease that of propionate. Considering that glycerol which is one of the main products in lipid metabolism could be fermented into propionate, butyrate, valerate, and isovalerate rather than acetate [32], LPL supplementation might affect glycerol fermentation pathway in the rumen. Few studies have investigated the effects of LPL on the characteristics of rumen fermentation. Jenkins et al [7] reported that increasing levels of purified phospholipids (0, 10, 20, and 30 mg) increased the proportion of propionate in a linear manner without a negative effect on the total VFA during *in vitro* fermentation. Sontakke et al [8] observed no significant changes in the production of acetate, propionate, and butyrate *in vitro* when LPL extracted from rice bran was supplemented up to 10% in the ration. Lee et al [9] observed an increased proportion of valerate and decreased proportions of acetate and A:P ratio in dairy cattle. Lee et al [9] also reported that the effect of LPL can vary depending on the different products of LPL, because the proportion of LPL differs based on the sources and enzymatic hydrolysis of phospholipids. However, the discrepancy in individual VFAs between the findings of Lee et al [9] and those of the present study is difficult to explain, because the same LPL product was used. This may be partially explained by the higher dosage of LPL in the current study (HLPL, 0.16% of DM) compared to that used by Lee et al [9] (HLPL, 0.075% of DM). In addition, we supplemented a high dosage of corn oil in the forage diet, which might have influenced the effects of LPL on rumen fermentation.

Supplementation of LPL significantly reduced the relative proportion of lipid-utilizing bacteria (*A. lipolytica* and *B. proteoclasticus*), which might decrease lipid digestion (hydrolysis and biohydrogenation) in the rumen. This finding is similar to that of Rico et al [33] who reported that lysolecithin may inhibit the hydrolysis and biohydrogenation of the lipid source in the rumen. Polycarpo et al [34] observed that supplementation of LPL could decrease the count of gram-positive cocci in the jejunum of broilers when fed a corn-based diet containing beef tallow. These authors also speculated that LPL might exert an antimicrobial effect on the gram-positive microorganisms by changing the cell membrane permeability. In the present study, LPL supplementation negatively affected the proportions of several gram-positive bacteria (*R. albus*, *R. flavefaciens*, and *B. proteoclasticus*), as well as gram-negative bacteria (*F. succinogenes* and *A. lipolytica*), whereas the total bacterial counts tended to increase in a linear manner. Therefore, it is thought that LPL supplementation could be effective as an antimicrobial agent rather than as an emulsifier in the rumen.

## CONCLUSION

In this study, LPL supplementation had antimicrobial effects on cellulolytic (*F. succinogenes* and *R. albus*) and lipolytic (*A. lipolytica* and *B. proteoclasticus*) bacteria, with no significant difference in nutrient degradability (DM and NDF). Therefore, use of LPL may be an effective antimicrobial agent for ruminant animal which needed high energy diet. Further studies are needed to evaluate the effects of LPL supplementation on lipid utilization (hydrolysis and biohydrogenation) in the rumen and availability of a lipid source in the small intestine.

## Figures and Tables

**Table 1:** Estimated chemical composition (% dry matter basis) of experimental diets used *in vitro*

Items	Treatments1)			

	CON	OiL	LLPL	HLPL
DM	94.80	94.46	94.46	94.46
CP	1.77	1.65	1.65	1.65
NDF	75.84	70.53	70.50	70.47
ADF	48.36	44.98	44.96	44.94
Lignin	7.65	7.11	7.11	7.11
EE	1.07	7.99	7.99	7.99
Ash	6.12	5.69	5.69	5.68
Ca	0.30	0.27	0.27	0.27
P	0.08	0.08	0.08	0.08
NFC	13.67	12.71	12.75	12.78

DM, dry matter; CP, crude protein; NDF, neutral detergent fiber analyzed with heat-stable α-amylase; ADF, acid detergent fiber; EE, ether extract; NFC, non-fibrous carbohydrate; LPL, lysophospholipids.

1) CON, annual ryegrass; OiL, 93% annual ryegrass + 7% corn oil on a DM basis (an experimental substrate); LLPL, an experimental substrate with 0.08% DM of LPL; HLPL, an experimental substrate with 0.16% DM of LPL.

**Table 2:** Polymerase chain reaction primers used in this study

Target species	Primer	Sequence (5′ → 3′)	Size (bp)	Efficiency1)	References
General bacteria	F	CGGCAACGAGCGCAACCC	130	1.90	[19]
	R	CCATTGTAGCACGTGTGTAGCC			
Ciliate protozoa	F	GCTTTCGWTGGTAGTGTATT	223	1.89	[35]
	R	CTTGCCCTCYAATCGTWCT			
Fungi	F	GAGGAAGTAAAAGTCGTAACAAGGTTTC	120	2.10	[19]
	R	CAAATTCACAAAGGGTAGGATGATT			
*Fibrobacter succinogenes*	F	GTTCGGAATTACTGGGCGTAAA	121	1.91	[19]
	R	CGCCTGCCCCTGAACTATC			
*Ruminococcus albus*	F	CCCTAAAAGCAGTCTTAGTTCG	176	2.02	[36]
	R	CCTCCTTGCGGTTAGAACA			
*Ruminococcus flavefaciens*	F	CGAACGGAGATAATTTGAGTTTACTTAGG	132	1.88	[19]
	R	CGGTCTCTGTATGTTATGAGGTATTACC			
*Butyrivibrio fibrisolvens*	F	ACCGCATAAGCGCACGGA	65	1.85	[37]
	R	CGGGTCCATCTTGTACCGATAAAT			
*Butyrivibrio proteoclasticus*	F	TCCGGTGGTATGAGATGGGC	185	2.07	[38]
	R	GTCGCTGCATCAGAGTTTCCT			
*Anaerovibrio lipolytica*	F	TGGGTGTTAGAAATGGATTC	597	1.88	[39]
	R	CTCTCCTGCACTCAAGAATT			

bp, base pair.

1) Efficiency is calculated as [10^−1/slope^].

**Table 3:** Gas production parameters after *in vitro* incubation of experimental diets using strained rumen fluid

Items	Treatments1)				SEM	p-value2)		
	
	CON	OiL	LLPL	HLPL		TRT	OiL-LPL	Lin
Gas (mL/g DM)								
3 h	29.8	27.6	27.7	28.2	0.95	0.155	0.805	0.921
6 h	45.4	42.9	42.8	42.9	1.08	0.119	0.936	0.972
12 h	74.8^a^	70.0^b^	69.5^b^	70.2^b^	1.07	0.004	0.367	0.484
24 h	129.0	119.5	119.9	122.8	3.08	0.052	0.700	0.912
48 h	199.8^a^	185.5^b^	187.1^b^	191.1^ab^	3.44	0.013	0.710	0.689
Fitted parameters of gas3)								
V_max_	267.7	246.0	252.6	259.9	9.32	0.193	0.964	0.493
K_g_	0.028	0.029	0.028	0.027	0.0015	0.834	0.924	0.564

SEM, standard error of the mean; DM, dry matter.

1) CON, annual ryegrass; OiL, 93% annual ryegrass + 7% corn oil on a dry matter (DM) basis (an experimental substrate); LLPL, an experimental substrate with 0.08% DM of lysophospholipids (LPL); HLPL, an experimental substrate with 0.16% DM of LPL.

2) TRT, treatment effect; OiL-LPL, OiL vs LPL treatment; Lin, a linear effect of LPL.

3) V_max_, theoretical maximum gas production (mL/g DM); K_g_, fractional rate of gas production (h^−1^).

a,b Values in the same row with different letters differ significantly (p < 0.05).

**Table 4:** Fermentation characteristics after *in vitro* incubation of experimental diets using strained rumen fluid

Items	Treatments1)				SEM	p-value2)		
	
	CON	OiL	LLPL	HLPL		TRT	OiL-LPL	Lin
IVDMD (%)	63.0	63.6	62.7	63.4	0.68	0.625	0.177	0.177
IVNDFD (%NDF)	61.3^a^	58.9^b^	58.3^b^	58.8^b^	0.65	0.007	0.315	0.310
pH	6.7	6.6	6.5	6.5	0.07	0.821	0.539	0.302
NH_3_-N (mg/100 mL)	38.3	37.7	37.9	37.9	0.99	0.951	0.876	0.829
TVFA (mM)	64.6	63.4	62.3	63.3	0.97	0.229	0.330	0.372
C2 (mmol/mol)	605.9^a^	589.5^b^	591.4^b^	593.5^b^	2.07	< 0.001	0.984	0.414
C3 (mmol/mol)	235.3^b^	251.4^a^	245.7^a^	245.5^a^	2.34	< 0.001	0.240	0.057
C4 (mmol/mol)	101.0^ab^	99.7^b^	102.2^a^	100.9^ab^	0.52	0.010	0.007	0.004
iso-C4 (mmol/mol)	11.1	11.0	11.0	10.9	0.09	0.151	0.170	0.641
C5 (mmol/mol)	26.4^b^	27.9^a^	28.9^a^	28.7^a^	0.38	< 0.001	< 0.001	< 0.001
iso-C5 (mmol/mol)	20.3^b^	20.5^ab^	20.8^a^	20.5^ab^	0.13	0.032	0.021	0.037
A:P ratio	2.6^a^	2.3^b^	2.4^b^	2.4^b^	0.03	< 0.001	0.363	0.105

SEM, standard error of the mean; IVDMD, *in vitro* dry matter degradability; IVNDFD, *in vitro* neutral detergent fiber degradability; NH_3_-N, ammonia nitrogen; TVFA, total volatile fatty acids; C2, acetate; C3, propionate; C4, butyrate; iso-C4, iso butyrate; C5, valerate; iso-C5, iso valerate; A:P ratio, acetate to propionate ratio.

1) CON, annual ryegrass; OiL, 93% annual ryegrass + 7% corn oil on a dry matter (DM) basis (an experimental substrate); LLPL, an experimental substrate with 0.08% DM of lysophospholipids (LPL); HLPL, an experimental substrate with 0.16% DM of LPL.

2) TRT, treatment effect; OiL-LPL, OiL vs LPL treatment; Lin, a linear effect of LPL.

a,b Values in the same row with different letters differ significantly (p < 0.05).

**Table 5:** Microbial abundance after *in vitro* incubation of experimental diets using strained rumen fluid

Items	Treatments1)				SEM	p-value2)		
	
	CON	OiL	LLPL	HLPL		TRT	OiL-LPL	Lin
Absolute abundance3)								
Total bacteria	4.7	3.9	4.3	4.2	0.44	0.318	0.120	0.089
Ciliate protozoa	8.2	6.2	7.5	7.2	1.41	0.502	0.409	0.252
Fungi	7.6	3.7	2.7	3.9	2.08	0.136	0.048	0.150
Relative proportion, % total bacteria								
*Fibrobacter succinogenes*	27.3^a^	19.0^ab^	9.0^b^	12.6^b^	4.14	0.008	0.094	0.028
*Ruminococcus albus*	10.4^a^	6.7^b^	5.3^b^	6.0^b^	0.51	< 0.001	0.011	0.006
*Ruminococcus flavefaciens*	0.29^a^	0.20^b^	0.17^b^	0.18^b^	0.020	0.001	0.178	0.102
*Butyrivibrio fibrisolvens*	0.37	0.34	0.34	0.29	0.034	0.208	0.573	0.933
*Anaerovibrio lipolytica*	0.13^c^	0.67^a^	0.48^b^	0.42^b^	0.035	< 0.001	0.099	0.003
*Butyrivibrio proteoclasticus*	13.1^b^	29.7^a^	16.6^b^	20.3^b^	2.91	0.002	0.024	0.003

SEM, standard error of the mean.

1) CON, annual ryegrass; OiL, 93% annual ryegrass + 7% corn oil on a dry matter (DM) basis (an experimental substrate); LLPL, experimental substrate with 0.08% DM of lysophospholipids (LPL); HLPL, experimental substrate with 0.16% DM of LPL.

2) TRT, treatment effect; OiL-LPL, OiL vs LPL treatments; Lin, linear effect of LPL.

3) Total bacteria, × 10^10^ copies/mL of rumen fluid; Ciliate protozoa, × 10^8^ copies/mL of rumen fluid; Fungi, × 10^6^ copies/mL of rumen fluid.

a,b Values in the same row with different letters differ significantly (p < 0.05).
